# Optical Coherence Tomography to Better Assess Chronic Total Occlusion Percutaneous Intervention Results: The PERFECTO Study

**DOI:** 10.1016/j.jacadv.2025.102125

**Published:** 2025-09-09

**Authors:** Sébastien Levesque, Benjamin Faurie, Benoit Lattuca, Julien Lemoine, Gael Pitchecanin, Pascal Motreff, Erwan Bressollette, Stéphanie Ragot, Claire Bouleti, Luc-Philippe Christiaens

**Affiliations:** a University Hospital of Poitiers, Poitiers, France; b Infirmerie Protestante de Lyon, Cardiology Department, Caluire-et-Cuire, France; c ACTION Study Group, Cardiology Department, Nîmes University Hospital, Montpellier University, Nîmes, France; d Clinique Louis Pasteur d'Essay les Nancy, Essey-lès-Nancy, France; e DACTIM: Data Analysis and Computation Through Imaging and Modeling- Mathematiques Images Sante/LMA/CNRS 7348, Hopital de La Rochelle, La Rochelle, France; f University Hospital of Clermont-Ferrand, Clermont-Ferrand, France; g Hôpital privé du Confluent, Nantes, France; h Centre d'Investigation Clinique, Inserm 1402, University Hospital of Poitiers, FACT Study Group, Poitiers, France

**Keywords:** chronic total occlusion, late-acquired malapposition, optical coherence tomography

## Abstract

**Background:**

Angioplasty of coronary chronic total occlusions (CTOs) was a breakthrough, but there is a lack of data concerning stent healing after these complex procedures.

**Objectives:**

The main aim of the PERFECTO (Post-stEnting assessment of Reendothelialization with optical Frequency domain imaging aftEr CTO procedure) study is to assess, for the first time, stent strut apposition at the index CTO procedure and at 3-month follow-up using frequency-domain optical coherence tomography (FD-OCT).

**Methods:**

From March 2018 to January 2020, 114 consecutive patients who underwent successful CTO recanalization >20 mm in length were prospectively included in 7 centers. FD-OCT was performed for ad hoc guidance during the index procedure and at 3-month follow-up. All patients received the same last-generation drug-eluting stent.

**Results:**

Mean age was 63.2 years, and 87% were male. The rate of malapposed struts per patient was 7.84% at the end of the index procedure and 15.03% at 3-month follow-up (*P* < 0.0001), highlighting the phenomenon of acquired malapposition. Malapposed struts occurred more often with dissection and re-entry techniques and subintimal stenting compared to intimal techniques (12.8% vs 5.3%, *P* = 0.02). At 3-month follow-up, distal vessel minimal lumen area increased from 69% (index 2.19 mm^2^ vs 3.71 mm^2^ at 3 months, *P* < 0.0001). No complication occurred with FD-OCT.

**Conclusions:**

CTO-percutaneous coronary intervention could affect stent healing with a high incidence of immediate and late-acquired malapposition. These results support the interest of using FD-OCT during follow-up to better assess CTO recanalization results. (Post-stenting Assessment of Reendothelialization With OFDI After CTO Procedure [PERFECTO]; NCT03209843)

The treatment of chronic total occlusion (CTO) of coronary arteries by percutaneous coronary intervention (PCI), although controversial, may lead to an improvement in quality of life, increased left ventricular ejection fraction, and a reduction of cardiac death rate compared to optimal medical treatment.^1^^,^^2^

Dissection and re-entry techniques (DART), such as antegrade dissection re-entry or reverse controlled antegrade and retrograde tracking (CART), use subintimal space for stenting and are aggressive techniques for the media artery in contrast to more conventional stenting using intimal techniques.^3^

The healing process of CTO lesions combined with the recanalization technique used is poorly investigated.

Frequency-domain optical coherence tomography (FD-OCT) has become the gold standard to identify coronary dissections, stent underexpansion, uncovered struts, and stent malapposition according to the recently published 1st Guidelines for FD-OCT PCI optimization^4^ and to improve immediate stent area.^5^^,^^6^

PERFECTO (Post-stEnting assessment of Reendothelialization with optical Frequency domain imaging aftEr CTO procedure) is the first study to systematically assess stent apposition both at index PCI and at 3-month follow-up.

The PERFECTO study was designed: 1) to compare strut malapposition between the index procedure and at a 3-month control; 2) to assess the impact of the crossing wire strategy (intimal vs subintimal techniques) on immediate and 3-month follow-up malapposition; and 3) to evaluate the safety of routine FD-OCT strategy in complex PCI.

## Methods

PERFECTO was an interventional, multicenter prospective study run in hospitals with a demonstrated expertise in CTO and FD-OCT (each selected center performed at least 50 CTO/y and 30 FD-OCT/y).

Conducted according to the ethical principles of the Declaration of Helsinki and the current guidelines for good clinical practice, this open-label trial was approved by the ethical committee and the Institutional Review Board OUEST III under the reference number 2016-A00867-44. Patients eligible for this study should present with a PCI of a CTO lesion >20 mm, a J-CTO score ≥1, and a suitable coronary artery for FD-OCT. Occlusion recanalization success probability is assessed by the J-CTO score, which uses 5 independent variables selected from the Japanese CTO registry, one of those being an occlusion length >20 mm.^7^ All patients signed an informed consent for this study. The authors are solely responsible for the design, the conduction, and all analyses of this study. The primary endpoint was the comparison of strut apposition using the FD-OCT at the index procedure and at 3-month follow-up. Secondary endpoints were to assess the impact of the crossing wire strategy (intimal vs subintimal techniques) on malapposition, the safety of FD-OCT strategy routine use, and systematic control in complex PCI (any procedural complication, fluoroscopy time, and amount of contrast used).

### Patients

Patient selection was angiography-based: PCI of a CTO lesion >20 mm in length (J-CTO score ≥1) and coronary artery anatomy suitable for FD-OCT. The indication of CTO-PCI was left to the physician according to guidelines.

The exclusion criteria were a failed PCI, the absence of written informed consent, the impossibility to perform a safe FD-OCT (major coronary tortuosity), pregnancy or child-bearing potential, a severe hemodynamic instability or severe chronic kidney disease defined by a creatinine clearance <30 mL/min, and severe coagulation disorders.

### CTO index PCI

All procedures were performed using the same new-generation thin-strut (80 μm), cobalt chromium, poly (DL-lactide co-caprolactone) biodegradable-polymer (15 μm), sirolimus-eluting stent Ultimaster (Terumo Corporation) to obtain comparable data regarding the healing process. The recommended inflated pressure was at least the nominal one (9 atm) during ≥30 seconds.

Successful crossing approaches for recanalization were classified as intimal techniques (antegrade or retrograde) and DART (antegrade dissection re-entry, retrograde, CART, and reverse CART).

### Index FD-OCT

After ensuring an optimal angiographic result and considering the procedure as if it was over, the operators immediately performed a systematic FD-OCT along the entire length of implanted drug-eluting stent, which could guide potential complementary revascularization maneuvers. All changes driven by FD-OCT results were reported on the electronic case report form. The OFDI Terumo Fastview (150 mm pullback at 1 time, pullback speed 5-40 mm/s, 100 frames per second) and the OCT Abbott Dragonfly Optis imaging catheter (54-74 mm pullback at 1 time, pullback speed 20 mm/s, 100 frames per second) were used in this study.

### Three-month follow-up

A coronary angiogram was systematically performed at 3 months using FD-OCT following the same method as for the index procedure (immediate FD-OCT analysis possibly followed by complementary therapies).

### FD-OCT analysis and endpoints

All angiograms and FD-OCT data were sent to the independent Corelab investigating center in Poitiers, France (LRCOM i3M-DACTIM-MIS team: Data Analysis and Computation Through Imaging and Modeling-Mathematics Images Sante/LMA/CNRS 7348) for postprocessing analysis. All the Corelab analyses were strut-based and not patient-based to allow for a more detailed description.

After calibration checking, the minimal lumen area (MLA) and minimal lumen diameter were measured from the semiautomatic contouring of the arterial lumen. Stent malapposition, or insufficient stent apposition, was defined by a distance between the center reflection of the strut and the vessel wall >400 μm, as previously published.^4^ An uncovered strut was considered as one without neointimal thickness. Malapposition was measured every 5 mm in the best cross-section.

### Statistical analysis

Continuous variables are reported as means with SDs or medians with IQRs according to the normality of the distribution, and categorical variables are reported as numbers and percentages of patients. Differences between groups were assessed using the Wilcoxon rank sum test for continuous variables (or Kruskall-Wallis test if more than 2 modalities), and the chi-square test or Fisher exact test when the expected cell value was <5 were used for categorical variables.

We evaluated the factors associated with stent reendothelialization and acquired stent malapposition at 3 months after CTO recanalization in univariate analyses, comparing index vs 3 months FD-OCT using Student's paired *t*-test or Wilcoxon matched-pair signed-rank test for quantitative variables or McNemar's chi-squared test for paired categorical variables. All statistical analyses were performed using SAS software (version 9.4, SAS Institute) by a senior statistician independent from the study (Prof. Stephanie Ragot, Clinical Investigation Center of Poitiers, Inserm 1402).

## Results

### Population

**Figure 1:**
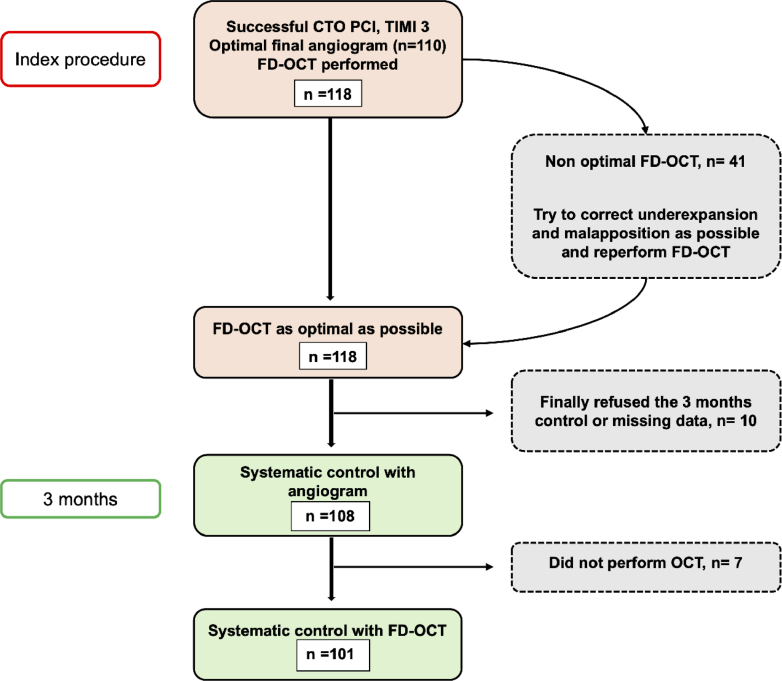
Flow Chart of the PERFECTO Study CTO = chronic total occlusion; FD-OCT = frequency-domain optical coherence tomography; OCT = optical coherence tomography; PCI = percutaneous coronary intervention; PERFECTO = Post-stEnting assessment of Reendothelialization with optical Frequency domain imaging aftEr CTO procedure.

A total of 124 patients undergoing CTO recanalization procedures were enrolled between March 7, 2018, and January 14, 2020 (Central Illustration). We excluded 6 patients owing to difficulties in the initial FD-OCT data collection (hard disk data erase). Finally, 118 patients were included in this study, of which 108 reached the 3-month follow-up. Of these 108 patients, 101 had an FD-OCT at the systematic 3-month control angiogram (2 reoccluded arteries, 5 technical FD-OCT problems) (Figure 1). The clinical characteristics of all 118 patients are detailed in Table 1. Mean age was 63 ± 12 years, and most patients (87%) were men. One-third were diabetics. Although a third of our population (34%) had severe coronary lesions (previous myocardial infarction), 42% of patients were still smokers.

**Central Illustration:**
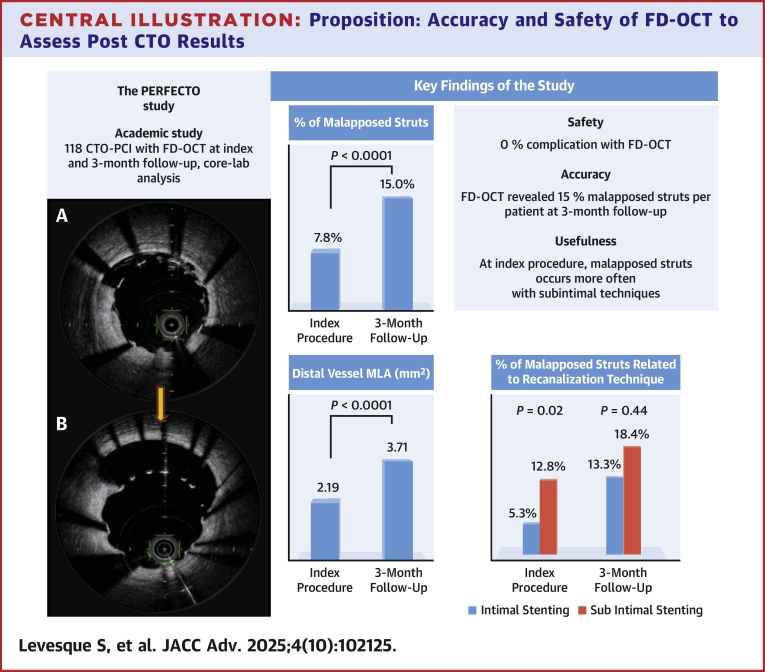
Proposition: Accuracy and Safety of FD-OCT to Assess Post CTO Results (A) Example of axial FD-OCT immediately after CTO-PCI “index procedure.” Deeply behind the struts, the media looks asymmetric: thinner on the top right quadrant than on the bottom left quadrant. Despite FD-OCT optimization, 2 struts remain malapposed. (B) Same-level axial FD-OCT at 3-month follow-up showing a late-acquired malapposition on the top right quadrant where the media looks the thinnest. Abbreviations as in Figures 1 and 2.

**Table 1:** Baseline Characteristics of the Population (N = 118)

Age (y)	63.2 ± 11.7
Male	103 (87)
Diabetes	35 (30)
Current smoking	49 (42)
Heredity	22 (19)
Dyslipidemia	85 (72)
Hypertension	70 (59)
Obesity (BMI >30 kg/m^2^)	27 (23)
Prior MI	40 (34)
Prior CABG	6 (5)
LVEF (%)	52.4 ± 12.7
Proof of viability	97 (82)
Angina status	
CCS I	83 (70)
CCS II	21 (18)
CCS III	12 (10)
CCS IV	2 (2)

Values are mean ± SD or n (%).

BMI = body mass index; CABG = coronary artery bypass grafting; CCS = Canadian Cardiovascular Society; LVEF = left ventricular ejection fraction; MI = myocardial infarction.

### Index lesions and procedural characteristics

The right coronary artery was affected by CTO in 75% of cases. Successful recanalization was mostly obtained by antegrade wiring (47.5%), while a retrograde or subintimal strategy was required in 16.1% and 36.4% of cases, respectively. The average stent length was 79.2 ± 26.9 mm. Procedural complications occurred in 7 (6%) cases (arrhythmia, n = 2; material stuck in a collateral branch, n = 2; occluded side branch, n = 1; contralateral dissection, n = 1; and vessel perforation resolved with covered stents, n = 1). The final angiographic analysis was considered optimal in 110 patients (93%); indeed, mechanical abnormalities such as residual dissection (n = 2), stent underexpansion (n = 4), or insufficient stent apposition (n = 2) were suspected in 8 (7%) patients after angiographic control (Table 2).

**Table 2:** Angiographic Data of Index CTO PCI (N = 118)

CTO fessel	
Left anterior descending	18 (15.3)
Right coronary artery	89 (75.4)
Left circumflex	11 (9.3)
Estimated length of occlusion (mm)	37.1 ± 21
Mean J-CTO score	2.4 ± 0.4
J-CTO score 0	0
J-CTO score 1	21 (17.8)
J-CTO score 2	47 (39.8)
J-CTO score 3	33 (30)
J-CTO score 4	15 (12.7)
J-CTO score 5	2 (1.7)
SYNTAX score	17 ± 8
Technique used for index procedure	
Antegrade wiring	56 (47.5)
Retrograde	19 (16.1)
CART	3 (2.5)
Reverse CART	19 (16.1)
Dissection and reentry	21 (17.8)
Estimated wire subintimal length (mm)	22 ± 25
Estimated wire subintimal length related to recanalization techniques (mm)	
True-to-true lumen technique: antegrade + retrograde	9.5 ± 14.9
Subintimal wiring technique: CART + reverse CART + dissection and re-entry	41.8 ± 24.1
Stent length (mm)	79 ± 27
Angiographic analysis post-PCI considered optimal	110 (93)
Procedural complication	7 (6)

Values are mean ± SD or n (%).

CART = controlled antegrade and retrograde tracking; CTO = coronary total occlusion; PCI = percutaneous coronary intervention.

### Index FD-OCT

The index FD-OCT read in the cath lab by the operator revealed abnormalities in 41 (35%) patients despite an angiographically optimal result in 93% of them (Table 3). Most abnormalities were stent underexpansion or malapposition (90%). Overall, 40 of the 41 (98%) patients with an FD-OCT-diagnosed abnormality received a complementary treatment: 35 patients needed a balloon dilatation, 3 an additional stenting, 1 patient received GP2b3a inhibitors, and 1 patient needed both additional stenting and AntiGP2b3a. When complementary treatment was applied, every patient had another FD-OCT control, which was centrally analyzed by the Corelab. The average additional contrast solution needed for the FD-OCT protocol was 40.4 mL (203 mL for the PCI procedure), and the additional X-ray fluoroscopic time was 1.92 minutes (40 minutes for the PCI procedure). No complication occurred during the protocol.

**Table 3:** FD-OCT Results and Safety at Index Procedure (N = 118)

Post-PCI FD-OCT considered as optimal	77 (65)
Post-PCI FD-OCT considered as nonoptimal	41 (35)
Malapposition	19 (16)
Underexpansion	18 (15)
Dissection	5 (4)
Thrombus	2 (2)
Others	2 (2)
Procedural changes induced by FD-OCT	40 (34)
Balloon postdilatation	35 (30)
Implantation of a new stent	4 (3)
GP2B3a use	2 (2)
Protocol safety	
Procedural complication related to FD-OCT protocol	0
Fluoroscopic time before FD-OCT (min)	39.8 ± 24.7
Additional fluoroscopic time for FD-OCT protocol (min)	1.9 ± 0.9
Contrast product before FD-OCT protocol (mL)	202.6 ± 87.4
Additional contrast product for FD-OCT protocol (mL)	40.4 ± 28.8

Values are mean ± SD or n (%).

FD-OCT = frequency-domain optical coherence tomography; PCI = percutaneous coronary intervention.

### The 3-month cath lab's follow-up

Despite their initial approval of the protocol design, the angiographic control was refused by 10 patients. Thus, angiographic analysis was performed in 108 patients, showing nonoptimal or suboptimal outcomes in 20 (18.6%) cases with 8 (7.4%) restenosis, 2 (2%) in-stent reocclusions, 6 (5.6%) dissections, and 7 (7%) significant distal stenosis, some patients having more than 1 complication (Table 4).

**Table 4:** Cathlab Analysis at 3-Month Follow-Up (N = 108)

Median follow-up (mo)	3 (2.9; 3.2)
All-cause death	0
Angiographic analysis	n = 108
Nonoptimal outcomes	20 (18.6)
Restenosis	8 (7.4)
Occlusion	2 (1.9)
Persistent distal stenosis	5 (4.6)
Persistent proximal stenosis	2 (1.9)
Dissection/hematoma	6 (5.6)
FD-OCT analysis	n = 101
No FD-OCT control	7
Stent thrombosis	2
Technical problems	5
Nonoptimal outcomes	48 (48)
Treated by balloon postdilatation	36 (36)
Treated by implantation of a new stent	6 (5.9)
Without additional procedure	11 (11)
Protocol safety	n = 101
Procedural complication per FD-OCT	0
Contrast volume use for FD-OCT (mL)	35 (17; 63)
Change in antiplatelet therapy or duration	32 (29)

Values are median (IQR) or n (%).

Abbreviation as in Table 4.

FD-OCT controls were impossible in 7 patients (2 due to stent thrombosis and 5 due to technical problems). FD-OCT was thus performed in 101 patients and revealed abnormalities in 48 (48%) patients, leading to immediate additional therapy in 42 patients (88%). These abnormalities justified an increased dual antiplatelet therapy (DAPT) time in 32 (29%) cases. The average contrast volume injected per FD-OCT protocol was 35 mL. No procedural complication occurred.

### Corelab analysis

**Figure 2:**
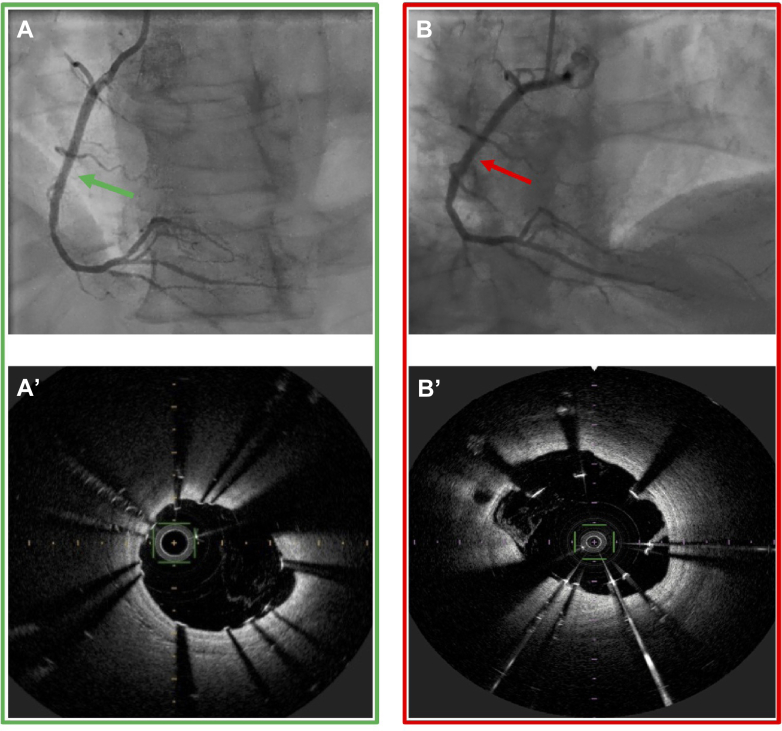
Example of Late-Acquired Malapposition (A) Angiogram of the right coronary artery immediately after recanalization at the index procedure. (A') FD-OCT axial view of the same right coronary artery immediately after recanalization at the index procedure (green arrow). Struts are all well apposed with a good stent deployment. MSA = 8.3 mm^2^ and MLA = 8.5 mm^2^. (B) Angiogram of the same right coronary artery at 3 months without obvious angiographic malapposition. (B') Same-level FD-OCT axial view as A' (red arrow; the level of axial view is identified with the collateral branch) at 3 months. While the MSA is quite the same, there are now 5 malapposed struts and an increased MLA of 9.5 mm^2^. FD-OCT = frequency-domain optical coherence tomography; MLA = minimal lumen area; MSA = minimal stent area.

Regarding the centralized reading of the index FD-OCT, as many as 84 patients had at least 1 malapposed strut (74%) (Tables 5 and 6). To differentiate between patients having only 1 malapposed strut and those with a high number of malapposed struts, we performed an analysis per strut. We analyzed 13,662 struts (stent length, 7,826 mm) at baseline and 14,326 struts (7588 mm) at 3 months, of which 1,120 (8.2%) and 2,068 (14.4%) were malapposed, respectively. Despite FD-OCT guidance and correction gestures, the rate of malapposed struts per patient at baseline remained considerable (7.84%). The significant increase in malapposed struts per patient from 7.84% at baseline to 15.03% at 3 months exposes late-acquired malapposition (*P* < 0.0001) (Table 5). These results are illustrated in Figure 2.

**Table 5:** Detailed FD-OCT Analysis Both at the Index and 3-Month Procedures

	Index FD-OCT	Intergroup Comparison for Index FD-OCT	3 Months FD-OCT	Intergroup Comparison for 3 Months FD-OCT	Intragroup Comparison Index vs 3 Months FD-OCT
Total number of analyzed struts, n = 101	13,662		14,326		
Total length of analyzed stents mm, n = 101	7,826		7,588		
Mean length of analyzed stents/patient (mm)	77.5 ± 27.1		77.4 ± 27.5		0.27
Minimal lumen area (mm^2^)	7.2 ± 1.8		7.8 ± 2.0		<0.0001
MLD at 0-5 mm distal to the stent (mm)	1.9 ± 0.4		2.2 ± 0.5		<0.0001
MLA at 0-5 mm distal to the stent (mm^2^)	2.19 ± 0.78		3.71 ± 0.91		<0.0001
Malapposition data, n = 101					
Number of patients with malaposition					0.0013
Without any malapposition	27 (27)		9 (9)		
With at least 1 malapposition	74 (74)		92 (92)		
Number of malapposed/analyzed struts	1,120/13,662		2068/14,326		
Malapposed struts/patient (%)	7.8 ± 10.7		15.0 ± 14.3		<0.0001

Values are mean ± SD or n (%).

FD-OCT = frequency-domain optical coherence tomography; MLA = minimal lumen area; MLD = minimal lumen diameter.

**Table 6:** FD-OCT Stratified Analysis at the Index and 3-Month Procedures

n = Number of Patients	Index FD-OCT	Intergroup Comparison for Index FD-OCT	3 Months FD-OCT	Intergroup Comparison for 3 Months FD-OCT	Intragroup Comparison Index vs 3 Months FD-OCT
Malapposed struts/patient related to recanalized artery (%)		0.0737		0.5789	
Left anterior descending artery (n = 16)	3.5 ± 4.4		14.3 ± 15.7		0.0171
Circumflex artery (n = 11)	8.8 ± 8.2		10.4 ± 7.8		0.4769
Right coronary artery (n = 74)	8.7 ± 11.8		15.9 ± 14.7		<0.0001
Malapposed struts/patient related to recanalization technique (%)		0.0953		0.8481	
Antegrade wiring (n = 49)	5.1 ± 5.7		12.8 ± 11.2		<0.0001
Retrograde (n = 18)	6.0 ± 4.8		14.6 (11, 12)		0.0042
CART (n = 3)	3.7 ± 2.2		13.3 ± 9.1		0.1088
Reverse CART (n = 15)	8.5 ± 7.5		16.9 ± 17.8		0.0995
Dissection and reentry (n = 16)	18.6 ± 20.6		20.8 ± 21.4		0.6791
Malapposed struts per patient related to recanalization technique summarized in 2 classes (%)		0.024		0.4419	
True-to-true lumen techniques: antegrade + retrograde (n = 67)	5.3 ± 5.5		13.3 ± 11.1		<0.0001
Subintimal wiring techniques: CART + reverse CART + dissection and re-entry (n = 34)	12.8 ± 15.8		18.4 ± 18.8		0.0768
Malapposed struts per patient related to subintimal wiring length (%)		0.4817		0.8373	
<10 (n = 34)	6.6 ± 6.3		15.6 ± 12.5		0.0001
10-20 (n = 20)	5.0 ± 4.7		12.4 ± 8.8		0.004
≥20 (n = 39)	11.3 ± 15.2		17.3 ± 18.2		0.034
Malapposed struts per patient related to J-CTO score (%)		0.3604		0.2398	
<2 (n = 17)	5.1 ± 6.9		10.8 ± 11.4		0.0557
2 (n = 42)	8.5 ± 12.9		13.6 ± 10.6		0.0027
≥3 (n = 39)	8.5 ± 9.8		18.5 ± 18.2		0.0008

Values are mean ± SD or n (%).

Abbreviations as in Table 2.

Malapposition occurred more often at baseline with DART and subintimal stenting vs intimal stenting (12.82% vs 5.32%, *P* = 0.024) but at 3-month follow-up, there was no significant difference between those groups (*P* = 0.440).

Patients with a J-CTO score ≥2 had a significant increase of malapposed struts between baseline and 3-month follow-up (from 8.51% to 13.57%, *P* = 0.003 for J-CTO = 2 group; from 8.54% to 18.45%, *P* = 0.0008 for the J-CTO ≥3 group), while the difference did not reach significance for J-CTO score <2 (*P* = 0.056) (Table 6).

The mean MLA of the stent-part artery increased from 7.2 to 7.8 mm^2^ (8% increase, *P* < 0.0001). We also found a vessel expansion of the nonstent distal part (distal measure 0-5 mm after the last axial FD-OCT images showing stent struts) with MLA increasing from 2.19 mm^2^ at baseline to 3.71 mm^2^ at 3 months (69% increase, *P* < 0.0001). The mean minimal lumen diameter in the same condition increased from 1.9 to 2.2 mm (15% increase, *P* < 0.0001).

## Discussion

To our knowledge, PERFECTO is the first study applying a routine FD-OCT at index PCI and at 3-month follow-up after CTO recanalization.

The major findings of this study are:

1. The late-acquired malapposed struts phenomenon was demonstrated with a significant increase of malapposed struts per patient at 3 months (15.03% as compared with baseline 7.84%, *P* < 0.0001).
2. Immediate malapposed struts occur more often at the end of index CTO-PCI with subintimal techniques.
3. An improved distal MLA expansion (69%) was observed at 3 months after successful recanalization.
4. Safety and accuracy of FD-OCT controls even in extreme PCI without any complication.

### Immediate malapposition and risk of late-acquired malapposition

The major finding of our study is the demonstration of the late-acquired malapposition phenomenon since patients benefited from an FD-OCT both at the index procedure and at the 3-month follow-up. Late malapposition was described in the ISAR-OCT-CTO study after subintimal recanalization strategy in 13.6% of cases and after intraplaque recanalization strategy in 6.6%, but without any OCT control at the index procedure. Thus acquired, or persistent malapposition could not be demonstrated.^8^ Malapposition is considered as a possible predictive factor of stent thrombosis and cardiovascular outcomes. In the PESTO registry, stent thromboses were associated with a mechanical abnormality in 96% of cases, mostly malapposition.^9^ Since diameter stenosis and calcified, long lesions are independent predictors of stent malapposition,^10^ CTO would be the perfect substrate for these mechanical abnormalities. In the AngiographiC Evaluation of the Everolimus-Eluting Stent in Chronic Total Occlusions (ACE-CTO) study, Sherbet et al found a 9.2% rate of malapposed strut after CTO-PCI.^11^ Our results confirm a high incidence of immediate malapposition (7.84%) even after FD-OCT-guided correction. These results are higher than previous studies in non-CTO patients, which detected 0.5% to 2.9% of malapposition.^11^

There are several explanations for the high rates of stent strut malapposition after CTO stenting. Indeed, due to negative remodeling, the lumen area increases after recanalization. Additionally, a calcified vessel wall causes mechanical stresses on the stent structure. Finally, successful revascularization of such lesions often requires a subintimal wire position and distal re-entry with disruption of the vessel integrity with formation of an intramural hematoma and stent implantation in the subintimal space, within the subintimal layer of the vessel wall^12^ and stenting of the false lumen results in stent malapposition.^8^

### Distal luminal diameter

Angiography performed immediately after CTO recanalization might underestimate the size of the distal vessel. The distal luminal area of the CTO vessel increases significantly after recanalization at 3 months (+69%, *P* < 0.001). As shown previously after CTO-PCI, Galassi et al measured the luminal diameter at 5 mm from the distality of the stent and found 2.0 ± 0.52 mm at post-PCI and 2.25 ± 0.50 mm at follow-up (*P* < 0.001).^13^ In 91 CTO patients, Gomez-Lara et al showed notable lumen and vessel enlargement. This might be caused by chronic hypoperfusion, potentially leading to stent-vessel mismatch and acquired malapposition.^14^

### Safety and clinical implications

The accuracy and safety of FD-OCT in PCI guidance has already been reported.^15^ We confirm here the safety of FD-OCT for CTO lesions, as we observed no procedural complication related to FD-OCT.

The high rate of acquired malapposition suggests the benefit of an angiographic and FD-OCT control to obtain better long-term results as suggested in the recent ILUMIEN IV and OCTOBER studies.^5^^,^^16^ A high rate of postdilatation needs to be anticipated with the routine use of stent platform allowing an oversizing expansion diameter of 15%.

DAPT duration is still one of the most important therapeutic implications regarding the risk of stent thrombosis. The high frequency of malapposition found at 3 months after CTO-PCI could lead practitioners to extend the DAPT duration, although the long-term (>1 year) benefits of prolonged DAPT have not been demonstrated in retrospective cohort studies.^17^

### Study Limitations

This study was a nonrandomized multicenter study, given the impossibility of performing a randomized study. Although one of the largest FD-OCT CTO guidance series, the number of subjects remains limited to obtain hard clinical evidence. Therefore, no clinical impact was assessed in this study, which focused on malapposition after CTO procedures as evaluated by FD-OCT from a technical perspective. Finally, the exact stent expansion rate could not be provided because the OCT software available at the time of this study did not offer automated assessment of stent expansion. Moreover, given the extensive stent lengths per patient, substantial discrepancies between proximal and distal segment sizes would likely have been observed.

## Conclusions

Findings of the PERFECTO study showed that complex vascular remodeling of chronically occluded vessels can increase the rate of acquired stent malapposition. The use of angiography and FD-OCT control at 3-month follow-up confirmed its safety and interest in detecting stent abnormalities.

Perspectives **COMPETENCY IN MEDICAL KNOWLEDGE:** The systematic FD-OCT after CTO lesion recanalization brought to light a high rate of stent strut malapposition after the index procedure and even more during follow-up (acquired malapposition). This may be explained by a negative remodeling as evidenced by the significant increase in distal lumen area, leading to stent-vessel mismatch. Given that CTO procedures are complex and associated with stent malapposition, known to be a factor of stent thrombosis, a systematic FD-OCT control could improve the long-term results of these patients by complementary gestures to optimize stent apposition. Moreover, in case of malapposition, DAPT duration could be extended to prevent the risk of stent thrombosis.

**TRANSLATIONAL OUTLOOK:** Future work with FD-OCT could help refine the diagnosis of malapposition, with a more detailed classification than just a binary criterion. Larger prospective studies are needed to confirm the potential clinical implications of FD-OCT control after CTO revascularization.

## Funding support and author disclosures

The main funding source is the Alienor Fundation of the University Hospital of Poitiers (https://www.fonds-alienor.fr/interview-du-dr-sebastien-levesque/). Dr Lattuca has received research grants from Biotronik, Boston Scientific, and the 10.13039/501100020699Institute of CardioMetabolism and Nutrition and lecture fees from Abbott, AstraZeneca, Bayer, Medtronic, Novartis, Sanofi, and Terumo. Dr Levesque is a consultant for Asahi and Boston Scientific and has received lecture fees from Shockwave Medical. Dr Motreff is a consultant for Terumo and Abbott. Dr Faurie is consultant for Asahi, Boston, and Teleflex. All other authors have reported that they have no relationships relevant to the contents of this paper to disclose.
